# Child Life under Queen Victoria

**Published:** 1897-04-17

**Authors:** 


### Child Life under Queen Victoria.

### X.—THE LAW AND EDUCATION.

It would not be fair to conclude our remarks on what has been done for the benefit of children under the present reign, without touching on a law which, without being directly philanthropic in its intention, has had the most directly beneficent results. The law which compels every child to be educated up to a certain standard and age, would have rendered impossible many of the wrongs which we have touched on in previous articles. But the idea that education is the right of every child is still a comparatively new one.

In the pre-Reformation days the Church had its schools, and when these were destroyed private beneficence planted schools in many places. But these endowments, whether so intended or not, soon became absorbed by the middle and upper classes. For the poor there was the occasional bequest of money to be spent on apprenticing children to some craft; that is, a little technical training was considered good for them, but intellectual development was held to be unnecessary. Strange to say, it is in the Factory Acts that we find the germ of free education, the first Act, that of Sir Robert Peel in 1802, insisting that the "three R's" should be taught to the factory apprentices during some part of every day.

The State did not interfere in education at all till 1832, when a sum of £20,000 was voted for the purpose of aiding local effort in building schools. In England this fund was administered by the two great societies; in Scotland, where a parochial system of education had existed for centuries, by the minister and kirk-session of the various parishes. As yet, however, the State was only a contributor to education, not an educator, and the distribution of schools, as well as their quality, was unequal in the highest degree. The teachers were often uneducated men, too sickly or too stupid to earn a living in another way, or old women whose establishments resembled a *crèche* rather than a school. Therefore, in 1839 it was found necessary not merely to increase the grant to £30,000, but to institute an Education Department to control its distribution and inspect the work done.

The first essential to good schools is good teachers. The Government desired to establish a National Training College, but the jealousies we have spoken of compelled it to leave this task to the denominational societies. The policy of the department has been always, in the words of the author of "The State and Education,"* "to act with strict neutrality as between recognised religious communions, but to conduct its operations through their medium." The establishment of pupil teachers in the schools provided students for the colleges, which sent them back to the schools as trained teachers. Thus a higher standard of efficiency was gradually introduced into the teaching staff. But the fundamental question of school supply remained unsettled. Up to this time Government subsidies were given only in addition to the sums raised by local benevolence and in proportion to them. The result of this was that in poor localities where local charity did little, Government aid—most needed there—was least lavishly given. The next step in advance was taken by Lord John Russell in 1853, when he sought to give to towns of 5,000 inhabitants or over a power of self-rating, with a view to supplementing the income of existing schools. This proposal was rejected, but instead there was introduced the principle of giving a grant to the managers of schools where the children had completed a certain number of attendances, and where at least three-fourths of the pupils had been presented for inspection.

These innovations did much for the schools, even if chiefly by showing how much remained to do. Inspection showed very often the weakness of tuition. Here, for example, is the written reply to a question given by a child of average intelligence in 1855:—

"My duty toads God is to bleed in Him, to fering and to loaf withold your arts, withold my mine, withold my sold, and with my seruth, to whirchp and give Thanks, to put my old trash in Him, to call upon Him, to onner His old name and His world, and to save Him truly all the days of my life's end."

An appreciation of the moral and intellectual value of such answers led, in 1861, to the institution of the principle of "payment by results." Much has been said against this system, but something of the sort was inevitable. It is obvious that money could not be given by the State to schools which produced no results or unsatisfactory ones, and though at first the complaints of over-pressure and comparisons to the bed of Procrustes were not altogether unfair, the system is at present so modified as to do little injustice to either teacher or scholar. Payment by results might limit the intellectual activity of the best schools, but it excited intellectual activity in the hitherto inferior ones.

It is to be noted that up to this time education was not compulsory. The first great step towards this was taken by the Act of 1870—the Act by which School Boards came into being. School Boards were by no means universal. Where voluntary effort was sufficient for all reasonable needs, it was not necessary to establish a body with power to raise money for education from the rates; but where they were instituted they had powers assigned to them which have affected, more, perhaps, than was foreseen, the course of national education. Their power of raising money enabled them to build good schools, and appoint good teachers; and these good, well-appointed, well-taught Board schools killed out a large number of those insanitary, ill-taught, adventure schools in which education was a mere pretence. Then the Boards had power to compel attendance. Some parents pleaded poverty; and if the plea was held to be just, the Board could remit the fees. Indeed, it is obvious that you cannot compel a man to take a thing which he probably does not want, and to pay for it as well. Compulsory education meant in the long run free education, which is now within the reach of all. Additional grants from various sources have helped to plant free centres of higher and technical education throughout the country. Indeed, though the Queen's long reign has witnessed much legislation that has been more loudly acclaimed as beneficent, it has been marked by hardly any more likely to raise national character and ensure national prosperity than this slowly developing but now all-embracing scheme of national education.

* Henry Craik, Esq., C.B., LL.D., Secretary to the Scotch Education Department, to whose volume with the above title in Macmillan's "English Citizen" series the writer is indebted for the facts given in this article.

